# (5,10,15,20-Tetra­phenyl­porphyrinato-κ^4^ *N*)cobalt(II)–18-crown-6 (1/1)

**DOI:** 10.1107/S1600536810012080

**Published:** 2010-04-10

**Authors:** Anissa Mansour, Mohamed Salah Belkhiria, Jean-Claude Daran, Habib Nasri

**Affiliations:** a Département de Chimie, Faculté des Sciences de Monastir, Avenue de l’environnement, 5019 Monastir, Tunisia; b Laboratoire de Chimie de Coordination, CNRS UPR 8241, 205 route de Norbonne, 31077 Toulouse, Cedex 04, France

## Abstract

The asymmetric unit of the title compound, [Co(C_44_H_28_N_4_)]·C_12_H_24_O_6_, contains one half of a Co^II^(TPP) (TPP is tetra­phenyl­porphyrin) complex and one half of an 18-crown-6 mol­ecule of crystallization, both lying on inversion centers. The Co^II^(TPP) complex exhibits a nearly planar conformation of the porphyrinate core [maximum deviation = 0.069 (2) Å] with an average Co—N distance of 1.971 (4) Å. The distance between the Co atom and the closest O atom of the 18-crown-6 mol­ecule is 2.533 (2) Å, indicating a short non-bonded contact between the Co atom and the crown ether mol­ecule. An ethyl­ene group of the 18-crown-6 mol­ecule is disordered over two sites with occupancies of 0.565 (7) and 0.435 (7).

## Related literature

For general background to cobalt porphyrin species and their applications, see: Sanders *et al.* (2000 ▶). For the synthesis of Co(II) tetra­phenyl­porphyrin, see: Madure & Scheidt (1976 ▶). For related structures, see: Konarev *et al.* (2003 ▶, 2004 ▶); Nascimento *et al.* (2007 ▶); Smirnov *et al.* (1998 ▶); Lee *et al.* (2002 ▶); Iimura *et al.* (1988 ▶). For a description of the Cambridge Structural Database, see: Allen (2002 ▶). For the SIMU/ISOR restraints used in the refinement, see: McArdle (1995 ▶).
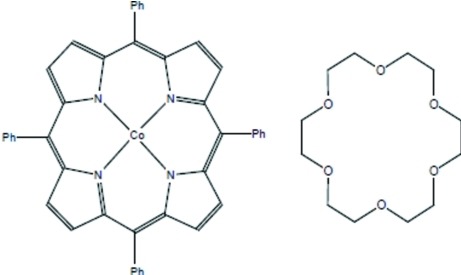


## Experimental

### 

#### Crystal data

[Co(C_44_H_28_N_4_)]·C_12_H_24_O_6_
*M* *_r_* = 935.95
Triclinic, 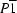

*a* = 10.1464 (4) Å
*b* = 11.0890 (6) Å
*c* = 11.7570 (5) Å
α = 104.327 (4)°
β = 105.842 (4)°
γ = 108.284 (4)°
*V* = 1125.12 (9) Å^3^
*Z* = 1
Mo *K*α radiation
μ = 0.44 mm^−1^
*T* = 180 K
0.25 × 0.24 × 0.21 mm

#### Data collection

Oxford Diffraction Xcalibur diffractometer
Absorption correction: multi-scan (*CrysAlis RED*; Oxford Diffraction, 2008 ▶) *T*_min_ = 0.927, *T*_max_ = 1.000
8862 measured reflections
4589 independent reflections
3977 reflections with *I* > 2σ(*I*)
*R*_int_ = 0.019

#### Refinement

*R*[*F* ^2^ > 2σ(*F* ^2^)] = 0.037
*wR*(*F* ^2^) = 0.100
*S* = 1.10
4589 reflections
323 parameters
30 restraints
H-atom parameters constrained
Δρ_max_ = 0.79 e Å^−3^
Δρ_min_ = −0.44 e Å^−3^

### 

Data collection: *CrysAlis CCD* (Oxford Diffraction, 2008 ▶); cell refinement: *CrysAlis RED* (Oxford Diffraction, 2008 ▶); data reduction: *CrysAlis RED*; program(s) used to solve structure: *SIR2004* (Burla *et al.*, 2005 ▶); program(s) used to refine structure: *SHELXL97* (Sheldrick, 2008 ▶); molecular graphics: *ORTEP-3 for Windows* (Farrugia, 1997 ▶); software used to prepare material for publication: *SHELXL97*.

## Supplementary Material

Crystal structure: contains datablocks I, global. DOI: 10.1107/S1600536810012080/pv2268sup1.cif

Structure factors: contains datablocks I. DOI: 10.1107/S1600536810012080/pv2268Isup2.hkl

Additional supplementary materials:  crystallographic information; 3D view; checkCIF report

## Figures and Tables

**Fig. 1.**
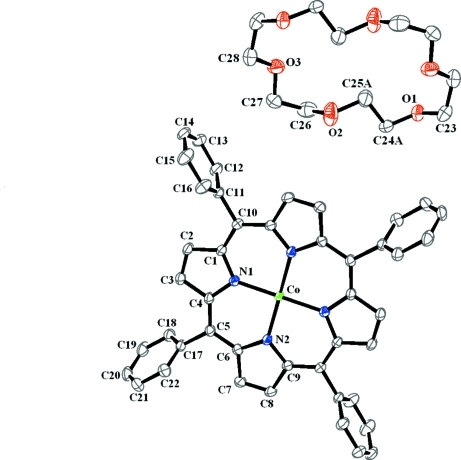
A view of the structure of (I), with the atom-numbering scheme. Unlabeled atoms are related by an inversion center to the labeled atoms. Displacement ellipsoids are drawn at 50%. The H atoms and the minor positions of the disordered C24 and C25 atoms have been omitted for clarity.

**Fig. 2.**
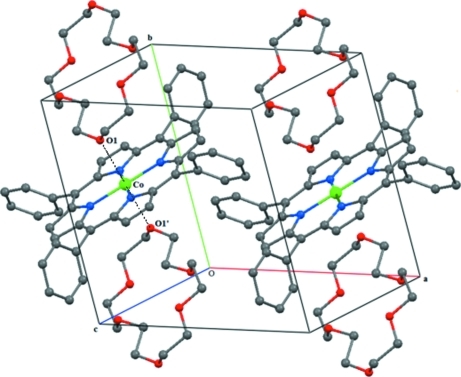
A unit-cell packing of (I). The short non-bonded contacts between the cobalt and the symmetry related O1 and O1' atoms of the two closest crown ether molecues, are drawn by dashed lines. The H atoms and the minor positions of the disordered C24 and C25 atoms have been omitted for clarity.

## supplementary crystallographic information

### Comment

Cobalt atom in metalloporphyrines is commonly used as a qualitatively acceptable substitute for iron atom in high-spin five-coordinate hemes of deoxyhemoglobin and in the low-spin oxygenated hemes of oxyhemoglobin. The metalation of a porphyrin by cobalt (using CoCl_2_^.^6H_2_O salt) yields the stable [Co^II^(Porph)] (Porph = porphyrin) complex used as starting material in the preparation of five and six-coordinated Co(II) and Co(III) metalloporphyrines (Sanders *et al.*, (2000). In the Cambridge Structural Database (CSD, version 5.31; Allen 2002) there are three structures of tetra-coordinated cobalt(II) tetraphenylporphyrin (TPP) complexes: IKUDOH (Konarev *et al.*, 2003), IXIKIJ (Konarev *et al.*, 2004) and TPORCP12 (Nascimento *et al.*, 2007). Herein we report the struture of the title compound,(I), which has been prepared in our laboratory.

The asymmetric unit of (I), contains one half [Co^II^(TPP)] complex and one half crystallographically independent 18-crown-6 molecule of crystallization both lying on inversion centers (Fig. 1).

The distance between the cobalt(II) ion and the symmetry related O1 and O1' atoms (Fig. 2) of the two closest crown ether molecules is 2.533 (2 ) Å. This distance is significantly longer than the Co^II^—O(THF) bond length [2.204 (4) Å] in the [Co^II^(F_8_TPP)(THF)_2_] derivative (where F_8_TPP is the tetrakis(pentafluorophenylporphyrin)) (Smirnov *et al.*, 1998) and the Co^II^—O(H_2_O) bond distance in the [Co^II^(H_2_O)_6_]^2+^ species [ 2.062 (4)- 2.141 (4) Å] (Lee *et al.*, 2002). This indicates that in (I) there is a short non-bonded contact between the cobalt ion and the crown ether molecule.

It has been noticed that there is a relationship between the ruffling of the porphyrinato core and the mean equatorial Co(II)—N_p_ distance (N_p_ = pyrrol N atom); the porphyrinato core is ruffled as the Co—N_p_ distance decreases, (Iimura *et al.*, 1988). Thus, the average distance Co—N_p_ in (I), 1.971 (4) Å, is longer than those of the three other reported [Co^II^(TPP)] structures quoted above [1.923 (3) – 1.969 (6) Å]. The porphyrin core in (I) presents a planar conformation with maximum and minimum deviations from the C_20_N_4_ least-squares plane of 0.069 (2) and -0.068 (2) Å for C6 and C8 atoms, respectively, while the Co^2+^ cation is basically in the porphyrin plane with a Co—C_t_ distance of 0.004 (1) Å (where C_t_ is the center of the C_20_N_4 _plane).

### Experimental

To a solution of [Co^II^(TPP)] (Madure & Scheidt 1976) (100 mg, 0.067 mmol) in chlorobenzene (10 ml) was added an excess of 18-crown-6 (150 mg, 0.567 mmol) and sodium methanolate (100 mg, 3.225 mmol). The reaction mixture was stirred at room temperature until reddisd-green solution was formed. The resulting material was crystallized by diffusion of hexanes through the chlorobenzene solution which yielded [Co^II^(TPP)]^.^(18-C-6) crystals as an impurity.

### Refinement

All H atoms were placed in geometrically idealized positions (C—H = 0.93-0.97 Å) and constrained to ride on their parent atoms, with *U*(H) = 1.2*U_eq_*(C). An ethylene group [C24 – C25] of the 18-crown-6 molecule was disordered over two sites with occupancies of 0.565 (7) and 0.435 (7). For this fragment, some anisotropic displacement ellipsoids were rather elongated which led us to use the SIMU/ISOR restraints (McArdle, 1995, Sheldrick, 2008). Alerts B and C for short intramolecular contacts H···H may be explained by the fact that C24 and C25 carbon atoms are disordered.

### Crystal data

**Table:**

[Co(C_44_H_28_N_4_)]·C_12_H_24_O_6_	*Z* = 1
*M**_r_* = 935.95	*F*(000) = 491
Triclinic, *P*1	*D*_x_ = 1.381 Mg m^−^^3^
Hall symbol: -P 1	Mo *K*α radiation, λ = 0.71073 Å
*a* = 10.1464 (4) Å	Cell parameters from 6796 reflections
*b* = 11.0890 (6) Å	θ = 3.1–32.2°
*c* = 11.7570 (5) Å	µ = 0.44 mm^−^^1^
α = 104.327 (4)°	*T* = 180 K
β = 105.842 (4)°	Prism, dark purple
γ = 108.284 (4)°	0.25 × 0.24 × 0.21 mm
*V* = 1125.12 (9) Å^3^	

### Data collection

**Table:**

Oxford Diffraction Xcalibur diffractometer	4589 independent reflections
Radiation source: fine-focus sealed tube	3977 reflections with *I* > 2σ(*I*)
graphite	*R*_int_ = 0.019
Detector resolution: 8.2632 pixels mm^-1^	θ_max_ = 26.4°, θ_min_ = 3.3°
ω and φ scans	*h* = −12→12
Absorption correction: multi-scan (*CrysAlis RED*; Oxford Diffraction, 2008)	*k* = −13→9
*T*_min_ = 0.927, *T*_max_ = 1.000	*l* = −14→14
8862 measured reflections	

### Refinement

**Table:**

Refinement on *F*^2^	Primary atom site location: structure-invariant direct methods
Least-squares matrix: full	Secondary atom site location: difference Fourier map
*R*[*F*^2^ > 2σ(*F*^2^)] = 0.037	Hydrogen site location: inferred from neighbouring sites
*wR*(*F*^2^) = 0.100	H-atom parameters constrained
*S* = 1.10	*w* = 1/[σ^2^(*F*_o_^2^) + (0.05322*P*)^2^ + 0.3421*P*] where *P* = (*F*_o_^2^ + 2*F*_c_^2^)/3
4589 reflections	(Δ/σ)_max_ < 0.001
323 parameters	Δρ_max_ = 0.79 e Å^−^^3^
30 restraints	Δρ_min_ = −0.44 e Å^−^^3^

### Special details

**Table:**

Geometry. All s.u.'s (except the s.u. in the dihedral angle between two l.s. planes) are estimated using the full covariance matrix. The cell s.u.'s are taken into account individually in the estimation of s.u.'s in distances, angles and torsion angles; correlations between s.u.'s in cell parameters are only used when they are defined by crystal symmetry. An approximate (isotropic) treatment of cell s.u.'s is used for estimating s.u.'s involving l.s. planes.
Refinement. Refinement of *F*\^2\^ against ALL reflections. The weighted *R*-factor wR and goodness of fit *S* are based on *F*\^2\^, conventional *R*-factors *R* are based on *F*, with *F* set to zero for negative *F*\^2\^. The threshold expression of *F*\^2\^ > σ(*F*\^2\^) is used only for calculating *R*-factors(gt) etc. and is not relevant to the choice of reflections for refinement. *R*-factors based on *F*\^2\^ are statistically about twice as large as those based on *F*, and *R*- factors based on ALL data will be even larger.

### Fractional atomic coordinates and isotropic or equivalent isotropic displacement parameters (Å^2^)

**Table:**

	*x*	*y*	*z*	*U*_iso_*/*U*_eq_	Occ. (<1)
Co	0.0000	0.5000	0.5000	0.01992 (11)	
N1	−0.10600 (16)	0.51602 (15)	0.34011 (13)	0.0205 (3)	
N2	−0.19511 (16)	0.39511 (15)	0.50211 (13)	0.0202 (3)	
C1	−0.04208 (19)	0.58671 (18)	0.27400 (16)	0.0207 (4)	
C2	−0.1554 (2)	0.57948 (19)	0.16553 (17)	0.0250 (4)	
H2	−0.1398	0.6187	0.1064	0.030*	
C3	−0.2885 (2)	0.50553 (19)	0.16529 (17)	0.0256 (4)	
H3	−0.3826	0.4838	0.1059	0.031*	
C4	−0.25852 (19)	0.46615 (18)	0.27385 (16)	0.0213 (4)	
C5	−0.36903 (19)	0.39375 (18)	0.30882 (16)	0.0217 (4)	
C6	−0.33699 (19)	0.36035 (18)	0.41648 (16)	0.0216 (4)	
C7	−0.4490 (2)	0.2741 (2)	0.44746 (18)	0.0268 (4)	
H7	−0.5525	0.2383	0.4042	0.032*	
C8	−0.3764 (2)	0.2551 (2)	0.55053 (18)	0.0276 (4)	
H8	−0.4199	0.2024	0.5917	0.033*	
C9	−0.21943 (19)	0.33090 (18)	0.58552 (16)	0.0217 (4)	
C10	0.1099 (2)	0.66163 (18)	0.30894 (16)	0.0213 (4)	
C11	0.15851 (19)	0.74003 (18)	0.23032 (16)	0.0224 (4)	
C12	0.1820 (2)	0.6798 (2)	0.12439 (18)	0.0293 (4)	
H12	0.1656	0.5883	0.1003	0.035*	
C13	0.2294 (2)	0.7541 (2)	0.05387 (19)	0.0339 (5)	
H13	0.2456	0.7125	−0.0169	0.041*	
C14	0.2529 (2)	0.8891 (2)	0.08771 (19)	0.0322 (4)	
H14	0.2848	0.9390	0.0401	0.039*	
C15	0.2289 (3)	0.9494 (2)	0.1922 (2)	0.0387 (5)	
H15	0.2439	1.0406	0.2153	0.046*	
C16	0.1824 (3)	0.8759 (2)	0.26378 (19)	0.0347 (5)	
H16	0.1672	0.9181	0.3349	0.042*	
C17	−0.52953 (19)	0.34897 (18)	0.22752 (17)	0.0229 (4)	
C18	−0.5921 (2)	0.2539 (2)	0.10469 (18)	0.0277 (4)	
H18	−0.5327	0.2183	0.0722	0.033*	
C19	−0.7417 (2)	0.2114 (2)	0.03001 (19)	0.0346 (5)	
H19	−0.7823	0.1476	−0.0522	0.042*	
C20	−0.8304 (2)	0.2635 (2)	0.0775 (2)	0.0371 (5)	
H20	−0.9312	0.2343	0.0279	0.045*	
C21	−0.7694 (2)	0.3590 (2)	0.1985 (2)	0.0372 (5)	
H21	−0.8289	0.3949	0.2304	0.045*	
C22	−0.6198 (2)	0.4018 (2)	0.27297 (19)	0.0308 (4)	
H22	−0.5793	0.4668	0.3545	0.037*	
O1	1.00507 (17)	0.72687 (17)	0.61845 (14)	0.0415 (4)	
O2	0.7723 (2)	0.8744 (2)	0.46275 (19)	0.0633 (5)	
O3	0.75479 (18)	0.98421 (16)	0.26483 (16)	0.0438 (4)	
C23	1.1145 (3)	0.8022 (2)	0.7438 (2)	0.0399 (5)	
H23A	1.1340	0.7388	0.7828	0.048*	
H23B	1.0739	0.8534	0.7930	0.048*	
C24A	0.8779 (5)	0.7518 (5)	0.5733 (4)	0.0333 (11)	0.565 (7)
H24A	0.8463	0.7817	0.6419	0.040*	0.565 (7)
H24B	0.7962	0.6691	0.5084	0.040*	0.565 (7)
C25A	0.9181 (5)	0.8618 (5)	0.5181 (5)	0.0465 (14)	0.565 (7)
H25A	0.9936	0.9474	0.5837	0.056*	0.565 (7)
H25B	0.9557	0.8353	0.4528	0.056*	0.565 (7)
C24B	0.9331 (11)	0.8256 (10)	0.5946 (9)	0.071 (2)	0.435 (7)
H24C	0.9026	0.8563	0.6639	0.085*	0.435 (7)
H24D	1.0063	0.9049	0.5921	0.085*	0.435 (7)
C25B	0.8162 (10)	0.7669 (8)	0.4875 (8)	0.075 (3)	0.435 (7)
H25C	0.7347	0.6982	0.4942	0.089*	0.435 (7)
H25D	0.8415	0.7234	0.4193	0.089*	0.435 (7)
C26	0.6814 (3)	0.8027 (2)	0.3354 (3)	0.0493 (6)	
H26A	0.5947	0.7262	0.3264	0.059*	
H26B	0.7366	0.7676	0.2905	0.059*	
C27	0.6318 (3)	0.8965 (3)	0.2809 (2)	0.0465 (6)	
H27A	0.5481	0.8445	0.1998	0.056*	
H27B	0.5997	0.9493	0.3378	0.056*	
C28	0.7410 (3)	1.1016 (2)	0.2499 (2)	0.0396 (5)	
H28A	0.7135	1.1454	0.3164	0.048*	
H28B	0.6623	1.0768	0.1687	0.048*	

### Atomic displacement parameters (Å^2^)

**Table:**

	*U*^11^	*U*^22^	*U*^33^	*U*^12^	*U*^13^	*U*^23^
Co	0.01575 (17)	0.0281 (2)	0.01649 (18)	0.00707 (14)	0.00525 (13)	0.01245 (14)
N1	0.0169 (7)	0.0242 (8)	0.0186 (7)	0.0062 (6)	0.0053 (6)	0.0095 (6)
N2	0.0202 (7)	0.0235 (8)	0.0168 (7)	0.0083 (6)	0.0053 (6)	0.0097 (6)
C1	0.0227 (9)	0.0237 (9)	0.0171 (8)	0.0096 (7)	0.0075 (7)	0.0100 (7)
C2	0.0272 (9)	0.0296 (10)	0.0191 (9)	0.0111 (8)	0.0067 (7)	0.0136 (8)
C3	0.0230 (9)	0.0310 (10)	0.0207 (9)	0.0104 (8)	0.0037 (7)	0.0120 (8)
C4	0.0204 (8)	0.0243 (9)	0.0177 (8)	0.0092 (7)	0.0043 (7)	0.0086 (7)
C5	0.0185 (8)	0.0234 (9)	0.0194 (8)	0.0072 (7)	0.0039 (7)	0.0073 (7)
C6	0.0173 (8)	0.0242 (9)	0.0199 (9)	0.0063 (7)	0.0050 (7)	0.0077 (7)
C7	0.0176 (8)	0.0329 (10)	0.0262 (9)	0.0054 (8)	0.0068 (7)	0.0132 (8)
C8	0.0220 (9)	0.0337 (10)	0.0260 (9)	0.0057 (8)	0.0097 (8)	0.0161 (8)
C9	0.0201 (8)	0.0248 (9)	0.0199 (9)	0.0070 (7)	0.0084 (7)	0.0098 (7)
C10	0.0244 (9)	0.0231 (9)	0.0187 (8)	0.0094 (7)	0.0095 (7)	0.0100 (7)
C11	0.0193 (8)	0.0274 (9)	0.0194 (8)	0.0066 (7)	0.0056 (7)	0.0124 (7)
C12	0.0378 (11)	0.0312 (10)	0.0279 (10)	0.0178 (9)	0.0161 (8)	0.0163 (8)
C13	0.0407 (12)	0.0461 (13)	0.0285 (10)	0.0223 (10)	0.0206 (9)	0.0215 (10)
C14	0.0288 (10)	0.0374 (11)	0.0275 (10)	0.0053 (9)	0.0080 (8)	0.0211 (9)
C15	0.0509 (13)	0.0244 (10)	0.0315 (11)	0.0065 (9)	0.0108 (10)	0.0123 (9)
C16	0.0486 (12)	0.0299 (11)	0.0245 (10)	0.0126 (9)	0.0159 (9)	0.0103 (8)
C17	0.0196 (8)	0.0249 (9)	0.0233 (9)	0.0071 (7)	0.0049 (7)	0.0138 (8)
C18	0.0258 (9)	0.0305 (10)	0.0242 (9)	0.0102 (8)	0.0063 (8)	0.0113 (8)
C19	0.0292 (10)	0.0346 (11)	0.0259 (10)	0.0033 (9)	−0.0010 (8)	0.0139 (9)
C20	0.0183 (9)	0.0468 (13)	0.0425 (12)	0.0075 (9)	0.0014 (8)	0.0290 (11)
C21	0.0258 (10)	0.0482 (13)	0.0492 (13)	0.0203 (10)	0.0163 (9)	0.0272 (11)
C22	0.0269 (10)	0.0347 (11)	0.0297 (10)	0.0121 (8)	0.0093 (8)	0.0122 (9)
O1	0.0360 (8)	0.0523 (10)	0.0342 (8)	0.0179 (7)	0.0067 (7)	0.0202 (7)
O2	0.0793 (12)	0.0724 (11)	0.0566 (10)	0.0555 (10)	0.0198 (9)	0.0282 (9)
O3	0.0461 (9)	0.0420 (9)	0.0594 (11)	0.0225 (8)	0.0307 (8)	0.0269 (8)
C23	0.0528 (14)	0.0367 (12)	0.0284 (11)	0.0177 (11)	0.0121 (10)	0.0137 (9)
C24A	0.032 (2)	0.036 (2)	0.035 (2)	0.0101 (18)	0.0153 (17)	0.0186 (18)
C25A	0.034 (2)	0.042 (3)	0.051 (3)	0.0053 (19)	0.0043 (19)	0.024 (2)
C24B	0.070 (3)	0.066 (3)	0.075 (3)	0.030 (2)	0.0147 (19)	0.035 (2)
C25B	0.074 (3)	0.068 (3)	0.080 (3)	0.035 (2)	0.0159 (19)	0.032 (2)
C26	0.0573 (15)	0.0349 (13)	0.0629 (17)	0.0176 (11)	0.0343 (13)	0.0181 (12)
C27	0.0293 (11)	0.0565 (15)	0.0486 (14)	0.0107 (10)	0.0121 (10)	0.0230 (12)
C28	0.0430 (13)	0.0389 (12)	0.0328 (11)	0.0201 (10)	0.0071 (10)	0.0104 (10)

### Geometric parameters (Å, °)

**Table:**

Co—N2	1.9669 (14)	C18—C19	1.385 (3)
Co—N2^i^	1.9669 (14)	C18—H18	0.9300
Co—N1^i^	1.9757 (14)	C19—C20	1.377 (3)
Co—N1	1.9757 (14)	C19—H19	0.9300
N1—C1	1.377 (2)	C20—C21	1.375 (3)
N1—C4	1.378 (2)	C20—H20	0.9300
N2—C9	1.377 (2)	C21—C22	1.383 (3)
N2—C6	1.379 (2)	C21—H21	0.9300
C1—C10	1.385 (2)	C22—H22	0.9300
C1—C2	1.430 (2)	O1—C24A	1.394 (4)
C2—C3	1.341 (3)	O1—C23	1.420 (3)
C2—H2	0.9300	O1—C24B	1.534 (8)
C3—C4	1.435 (2)	O2—C26	1.385 (3)
C3—H3	0.9300	O2—C25B	1.462 (7)
C4—C5	1.386 (2)	O2—C25A	1.510 (5)
C5—C6	1.388 (2)	O3—C28	1.399 (3)
C5—C17	1.490 (2)	O3—C27	1.413 (3)
C6—C7	1.434 (2)	C23—C28^ii^	1.488 (3)
C7—C8	1.339 (3)	C23—H23A	0.9700
C7—H7	0.9300	C23—H23B	0.9700
C8—C9	1.429 (2)	C24A—C25A	1.517 (6)
C8—H8	0.9300	C24A—H24A	0.9700
C9—C10^i^	1.386 (2)	C24A—H24B	0.9700
C10—C9^i^	1.386 (2)	C25A—H25A	0.9700
C10—C11	1.494 (2)	C25A—H25B	0.9700
C11—C12	1.380 (3)	C24B—C25B	1.308 (12)
C11—C16	1.381 (3)	C24B—H24C	0.9700
C12—C13	1.380 (3)	C24B—H24D	0.9700
C12—H12	0.9300	C25B—H25C	0.9700
C13—C14	1.373 (3)	C25B—H25D	0.9700
C13—H13	0.9300	C26—C27	1.493 (3)
C14—C15	1.370 (3)	C26—H26A	0.9700
C14—H14	0.9300	C26—H26B	0.9700
C15—C16	1.382 (3)	C27—H27A	0.9700
C15—H15	0.9300	C27—H27B	0.9700
C16—H16	0.9300	C28—C23^ii^	1.488 (3)
C17—C22	1.384 (3)	C28—H28A	0.9700
C17—C18	1.388 (3)	C28—H28B	0.9700
			
N2—Co—N2^i^	180.0	C20—C19—C18	119.9 (2)
N2—Co—N1^i^	90.63 (6)	C20—C19—H19	120.0
N2^i^—Co—N1^i^	89.37 (6)	C18—C19—H19	120.0
N2—Co—N1	89.37 (6)	C21—C20—C19	119.82 (18)
N2^i^—Co—N1	90.63 (6)	C21—C20—H20	120.1
N1^i^—Co—N1	180.0	C19—C20—H20	120.1
C1—N1—C4	104.78 (14)	C20—C21—C22	120.2 (2)
C1—N1—Co	126.92 (12)	C20—C21—H21	119.9
C4—N1—Co	128.22 (12)	C22—C21—H21	119.9
C9—N2—C6	104.48 (14)	C21—C22—C17	120.75 (19)
C9—N2—Co	126.91 (12)	C21—C22—H22	119.6
C6—N2—Co	128.44 (12)	C17—C22—H22	119.6
N1—C1—C10	125.90 (16)	C24A—O1—C23	122.1 (2)
N1—C1—C2	110.67 (15)	C23—O1—C24B	102.2 (4)
C10—C1—C2	123.35 (16)	C26—O2—C25B	97.5 (4)
C3—C2—C1	107.06 (16)	C26—O2—C25A	120.3 (3)
C3—C2—H2	126.5	C28—O3—C27	114.26 (18)
C1—C2—H2	126.5	O1—C23—C28^ii^	113.86 (18)
C2—C3—C4	107.11 (16)	O1—C23—H23A	108.8
C2—C3—H3	126.4	C28^ii^—C23—H23A	108.8
C4—C3—H3	126.4	O1—C23—H23B	108.8
N1—C4—C5	125.58 (16)	C28^ii^—C23—H23B	108.8
N1—C4—C3	110.38 (15)	H23A—C23—H23B	107.7
C5—C4—C3	123.97 (16)	O1—C24A—C25A	107.9 (3)
C4—C5—C6	122.88 (16)	O1—C24A—H24A	110.1
C4—C5—C17	118.89 (16)	C25A—C24A—H24A	110.1
C6—C5—C17	118.23 (16)	O1—C24A—H24B	110.1
N2—C6—C5	125.50 (16)	C25A—C24A—H24B	110.1
N2—C6—C7	110.62 (15)	H24A—C24A—H24B	108.4
C5—C6—C7	123.69 (16)	O2—C25A—C24A	104.3 (3)
C8—C7—C6	106.93 (16)	O2—C25A—H25A	110.9
C8—C7—H7	126.5	C24A—C25A—H25A	110.9
C6—C7—H7	126.5	O2—C25A—H25B	110.9
C7—C8—C9	107.22 (16)	C24A—C25A—H25B	110.9
C7—C8—H8	126.4	H25A—C25A—H25B	108.9
C9—C8—H8	126.4	C25B—C24B—O1	111.0 (8)
N2—C9—C10^i^	126.03 (16)	C25B—C24B—H24C	109.4
N2—C9—C8	110.74 (15)	O1—C24B—H24C	109.4
C10^i^—C9—C8	123.23 (16)	C25B—C24B—H24D	109.4
C1—C10—C9^i^	123.25 (16)	O1—C24B—H24D	109.4
C1—C10—C11	118.58 (15)	H24C—C24B—H24D	108.0
C9^i^—C10—C11	118.16 (16)	C24B—C25B—O2	106.7 (7)
C12—C11—C16	118.63 (17)	C24B—C25B—H25C	110.4
C12—C11—C10	121.28 (17)	O2—C25B—H25C	110.4
C16—C11—C10	120.09 (17)	C24B—C25B—H25D	110.4
C11—C12—C13	120.67 (18)	O2—C25B—H25D	110.4
C11—C12—H12	119.7	H25C—C25B—H25D	108.6
C13—C12—H12	119.7	O2—C26—C27	108.8 (2)
C14—C13—C12	120.34 (19)	O2—C26—H26A	109.9
C14—C13—H13	119.8	C27—C26—H26A	109.9
C12—C13—H13	119.8	O2—C26—H26B	109.9
C15—C14—C13	119.39 (18)	C27—C26—H26B	109.9
C15—C14—H14	120.3	H26A—C26—H26B	108.3
C13—C14—H14	120.3	O3—C27—C26	107.81 (19)
C14—C15—C16	120.54 (19)	O3—C27—H27A	110.1
C14—C15—H15	119.7	C26—C27—H27A	110.1
C16—C15—H15	119.7	O3—C27—H27B	110.1
C11—C16—C15	120.43 (19)	C26—C27—H27B	110.1
C11—C16—H16	119.8	H27A—C27—H27B	108.5
C15—C16—H16	119.8	O3—C28—C23^ii^	110.08 (18)
C22—C17—C18	118.42 (17)	O3—C28—H28A	109.6
C22—C17—C5	120.57 (17)	C23^ii^—C28—H28A	109.6
C18—C17—C5	121.01 (17)	O3—C28—H28B	109.6
C19—C18—C17	120.81 (19)	C23^ii^—C28—H28B	109.6
C19—C18—H18	119.6	H28A—C28—H28B	108.2
C17—C18—H18	119.6		

Symmetry codes: (i) −*x*, −*y*+1, −*z*+1; (ii) −*x*+2, −*y*+2, −*z*+1.
